# A Review of the Effects of Non-Straw Enrichment on Tail Biting in Pigs

**DOI:** 10.3390/ani9100824

**Published:** 2019-10-18

**Authors:** Stephanie Buijs, Ramon Muns

**Affiliations:** Agriculture Branch, Sustainable Agri-Food Sciences Division, Agri-Food and Biosciences Institute, Hillsborough BT26 6DR, UK; ramon.muns@afbini.gov.uk

**Keywords:** enrichment, straw, object, toy, tail biting, tail damage, manipulation, interaction, pig-directed, pen-directed

## Abstract

**Simple Summary:**

Tail biting, a damaging behaviour that one pig directs at another, causes pain, wounding and health problems. It reduces both pig welfare and market value. Enrichment can reduce tail biting substantially. Many pig producers are reluctant to use straw as enrichment, but many non-straw alternatives exist. We aimed to evaluate their ability to reduce tail biting based on studies on the effects of enrichment on tail damage and manipulation of other pigs, and on the duration of interaction with enrichment. Additionally, we reviewed how pigs interact with different enrichments (e.g., by rooting or chewing it). This was done to clarify which type of enrichment could satisfy which behavioural motivation (that may lead to tail biting if not satisfied). However, very little information on separate enrichment-directed behaviours was uncovered. Several effective types of non-straw enrichment were identified, but these correspond poorly with the types of enrichment commonly applied on commercial farms. More detailed observations of how pigs interact with different enrichments, other pigs, and their environment would improve our understanding of how to combine enrichments to minimize tail biting. This is essential because although single non-straw enrichments can reduce tail biting significantly, the remaining levels of damage can still be high.

**Abstract:**

Tail biting remains a common problem in pig production. As producers are reluctant to use straw to reduce this behaviour, we review studies on the effectiveness of other types of enrichment. Roughage, hessian sacks, compost, fresh wood, space dividers, rope, and providing new objects regularly can significantly reduce tail damage. These results should be interpreted with some caution, as often only one study per enrichment could be identified. No evidence was found that commonly applied enrichment objects (processed wood, plastic or metal) reduce tail biting significantly unless exchanged regularly, even though multiple studies per type of enrichment were identified. Many studies evaluated the duration of enrichment use, but few evaluated the manner of use. This hampers identification of combinations of enrichment that will satisfy the pig’s motivation to eat/smell, bite, root and change enrichments, which is suggested to reduce tail biting. New objects designed to satisfy specific motivations were shown to receive high levels of interaction, but their effectiveness at reducing tail damage remains unknown. More in-depth study of how pigs interact with non-straw enrichment, which motivations this satisfies and how this affects behaviour towards conspecifics, is necessary to optimize enrichment strategies. Optimization is necessary because ceasing tail docking in a way that improves pig welfare requires more effective enrichments than those described in this review, or alternatively, better control over other factors influencing tail biting.

## 1. Introduction

Tail biting is a damaging behaviour shown by pigs, which not only causes harm to the victims (e.g., painful tail lesions that can lead to infection), but also results in production losses (e.g., decreased growth and partial carcass condemnation due to infection) [1]. The problem has become more pressing due to the EU ban on tail docking (removal of part of the tail shortly after birth, which greatly reduces tail biting but is painful itself [2]). As the causation of tail biting is multifactorial, there are also many different strategies to reduce this behaviour [3]. Providing pigs with suitable environmental enrichment at the right time is one way to reduce tail biting as well as other welfare problems (e.g., insufficient opportunities for species-specific behaviour which leads to stress and knock-on effects on health) [4,5]. Opinions on enrichment’s importance relative to other tail biting reduction strategies differ both within and between the scientific and farming community. However, all parties agree that there is at least some merit to using enrichment to reduce this unwanted and damaging behaviour [3,4,6,7,8]. In this paper, we review existing knowledge on how enrichment affects tail biting in pigs, discussing effects on tail damage and behaviour directed towards conspecifics and the environment. We also investigate which motivation the different types of enrichment are likely to satisfy using information on how and how long pigs interact with these, as there is currently no consensus on how enrichment actually reduces tail biting.

**Figure 1:**
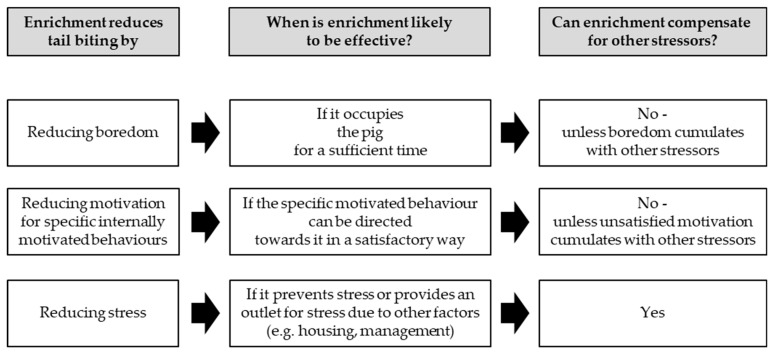
Potential mechanisms by which enrichment reduces tail biting and their consequences for the types of enrichment that are likely to be effective and interaction with other stressors.

Enrichment could reduce tail biting by reducing boredom or by providing opportunities to satisfy specific internal motivations for appetitive foraging [6,9] (Figure 1). In such cases, lack of stimulation due to absence of enrichment, would be expected to act independently of other stressors. However, a lack of stimulation has also been suggested to cumulate with other external stressors, and enrichment has been suggested to provide an outlet for stress or frustration caused by different aspects of housing and management [6,10,11]. The exact mechanism is of importance, as under the last option, enrichment could reduce the effect of factors in the environment that cause tail biting. Thus, enrichment would allow producers to make a rapid improvement to pig welfare in systems that are not optimally suited to prevent tail biting, but that cannot be changed easily themselves. 

Although the idea represented by the third pathway of Figure 1 (i.e., that enrichment decreases tail biting by providing an outlet for stress caused by a variety of factors) is common, there is little direct evidence for this. If enrichment alleviates or prevents stress due to other factors, this should lead to significant statistical interactions between enrichment and such factors (i.e., the effect size of the other factor would be smaller in the presence of enrichment). In line with this, one study found that increased stocking density [10] led to a smaller increase in pig-directed behaviour when enrichment was present. However, the majority of studies in which interactions between enrichment and other factors were modelled found that these interactions were not significant (e.g., [12,13,14,15]). This may mean that enrichment influences tail biting behaviour independently. Alternatively, studies may not have been able to estimate such interactions correctly due to insufficient statistical power to detect interactive effects. Note that an independent mechanism for enrichment does not mean that it is the only factor affecting tail biting (the multifactorial nature of the issue is well recognized [1]), just that enrichment does not influence the effects of these other factors on tail biting. 

In this review, we focus on non-straw enrichment as the beneficial effects of enriching pens with straw have already been shown many times [1,8,16], but the reluctance to apply straw in commercial pig farming remains. Reasons for this include the (potentially overestimated) risk of blockage of slurry systems [17], increased labour [18], poor local availability, a reduction of the pig’s capacity to lose heat by full body contact with the concrete floor [19], and concerns for biosecurity and food safety [5]. There are many alternatives to straw, however, some of which pigs actually prefer over straw (peat, compost, sand, sawdust, wood shavings, branches, bark, beets and silage [20,21]). Although the use of straw (or other loose organic materials) is common on commercial farms in some countries (Finland 72% of farms [22], United Kingdom 68% [23]) it is far less common in others (Germany 34% [24], Italy 0% [25]) where non-straw enrichments, including plastic objects, chains, rope, and wood, are much more common. Such non-straw enrichments are considered to be less effective, and to be sufficient only when provided in certain combinations. According to EU recommendations, the pig should be able to eat/smell, bite, root and change the location, appearance, or structure of this combination of enrichments [26]. But which specific types of enrichment adhere to each of these criteria is a matter of debate (e.g., whether anything that can be swallowed is to be considered edible, and whether investigation is to be limited to rooting, as suggested by the associated working document [27]). Determining a priori if an enrichment satisfies the four EU criteria is difficult as pigs have been reported to use enrichment in unexpected ways. Furthermore, predicting an enrichment’s use based upon its material characteristics and presentation is challenging: even when 27 characteristics were taken into account, less than 40% of the variation in enrichment use could be explained [28]. Experts show marked differences of opinion regarding the importance of specific characteristics, and differences between experts and meta-analysis of experimental studies have been noted as well [29]. Even when experts show reasonable agreement on which enrichments are most suitable, they show considerable differences when asked which enrichment is acceptable (i.e., suitable enough) [30]. 

## 2. Methods and Outline

The articles used for this review were retrieved through two separate searches on Web of Science. For the first search, we used the keywords ‘tail biting’ OR ‘tail-biting’ OR ‘tailbiting’ OR ‘tail damage’ for articles published between 2014 and 2019 (as part of a larger review project including multiple influences on tail biting). The second search was conducted using the keywords ‘enrichment’ AND ‘pig’. Publication year was not limited in this second search. Relevant articles (relating to non-straw enrichment and weaners, growers, fatteners, rearing gilts or piglets) were selected manually from the retrieved articles for both searches. Further relevant articles and EU legislation and guidelines were identified from the reference lists of the retrieved articles, during conference attendance, and by contacting government officials. 

Since we specifically wanted to review the effect of non-straw enrichment, studies were only included if the effect of the non-straw enrichment could be evaluated separately from the effect of straw. For the evaluation of tail damage and behaviour directed towards conspecifics and the environment, this meant that if straw was used as one of the experimental treatments, this treatment was discarded and only the treatments involving non-straw enrichment or no enrichment were included. If an equal amount of straw was provided in addition to each non-straw treatment, all of these treatments were included (as we assumed that the effect of the non-straw enrichment could be evaluated separately in such cases). In some studies evaluating the amount and type of interaction directed towards non-straw enrichment straw was present, but interaction with non-straw enrichment was measured separately from interaction with straw. These studies were included. Any studies using a single pen per evaluated enrichment were discarded, as we considered these to be insufficiently replicated to provide reliable evidence. Furthermore, data on tail damage was not included if it could not be separated from damage to other body parts (e.g., ears), as we specifically wanted to focus on damage to the tail. 

Several scoring systems have been used for tail damage, which vary in their exact definition and the number of severity categories included. For the purpose of this review, all tail damage data was classified into 3 categories when possible: no damage, mild damage (small bite marks, superficial bites or scratches, tail-end hair missing, or blood on the tail) and severe tail damage (partial or complete tail loss, crust formation, infection, fresh blood, or clearly visible wounds). Note that this was done to facilitate comparison between studies and that depending on which scoring system was used originally, damage categories may still differ slightly between studies. To compare studies that used different measures, we calculated the fold-changes (level of damage in enriched pens / level of damage in control pens) within each study [1].

The existing knowledge on non-straw enrichment’s capacity to reduce tail damage is discussed in Section 3 and effects on behaviours directed towards conspecifics and the environment in Section 4. In addition, we investigate which motivation the different types of enrichment are likely to satisfy using information on how much pigs interact with specific enrichments (Section 5) and the types of behaviour they direct at specific enrichments (Section 6).

## 3. Effects of Non-Straw Enrichment on Tail Damage 

A main consequence of tail biting behaviour is the damage it causes to the victim. Tail biting can result in severe inflammation beneath outwardly healthy skin [31], but usually damage ranges from missing hair, light bite marks or scratches to partial or complete loss of the tail [32,33]. For the purpose of this review, such signs have been reclassified into 3 categories when possible: no damage, mild damage and severe tail damage (see methods for details).

Table 1 shows that providing roughage [33], hessian fabric [34], compost [35], or freshly cut wood [32] preventively reduces the risk of tail damage significantly. Changing chewable enrichment objects weekly [36] and separating pen space by adding a wall or mezzanine floor [37] have also been shown to be effective. Adding a combination of objects (i.e., fresh wood, metal, plastic) reduced tail biting even in an already relatively enriched situation (i.e., straw, wood shavings) [32]. Even enrichment provided prior to weaning can reduce post-weaning tail damage [38]. Although all these non-straw enrichments were shown to be effective, this conclusion is usually based on a single study per enrichment material and should therefore be interpreted with caution. Furthermore, it needs to be emphasized that a significant reduction usually does not mean that no tail damage occurred. Severe tail damage occurred in 10–45% of undocked pigs in successfully enriched pens in several studies [32,33,38]. Although in some studies using undocked pigs tail damage was (nearly) absent [36,39], these studies also report little tail damage in the control treatment, or used a very high number of different enrichments. The occurrence of high levels of damage in successfully enriched pens emphasizes that enrichment is only one of many factors that influence tail damage [1,3], which is highly variable in nature. Moving away from tail docking in a way that improves pig welfare thus clearly necessitates enrichments that are more effective than those described in this review, or better control over the other factors that influence tail biting.

Providing pigs with blocks of pressed feed [40] or scattering feed [41] did not reduce tail damage significantly. Similarly, processed wood (posts, pressed shavings) or permanently present metal and plastic objects did not lead to a significant reduction in tail damage in any of the reviewed studies (although visual inspection of means sometimes suggested some decrease in damage) [32,35,40,42]. This is worrying, as such objects are the most common types of enrichment on many commercial farms [24,25]. Epidemiological studies on tail damage carried out on commercial pig farms in the UK and Italy could not show reduced tail damage in the presence of metal, plastic, and wooden objects [23,25]. Other objects have been designed more recently, specifically attempting to achieve more sustained interest from the pigs (e.g., by added scent or manipulation options), potentially making these more effective at reducing tail damage. Although there is some scientific knowledge on time spent in interaction with such objects (see Section 5), their ability to reduce tail damage remains to be tested. 

The EU has suggested several possible materials to be used to prevent tail biting [26]. The studies reviewed in this paper, indicating that roughage, compost, hessian sacks, fresh wood and, to a certain extent, rope effectively reduce tail damage, support some of the EU recommendations. However, for many other suggested materials, no studies on their ability to prevent tail damage when provided in the post-weaning phase could be identified (i.e., soil, wood shavings, sawdust, sand, stones, paper, and pellet dispensers). For another suggested enrichment (pressed sawdust), one study was identified but could not show a significant effect on tail damage [40], and although root vegetables were shown to reduce pen mate manipulation markedly, the direct effects on tail damage are unknown [43]. Another suggested material, wood, was only found to be effective when freshly cut [32], but not when posts were used [40]. In fact, these posts caused an increase in pen mate manipulation in two studies [14,44], although this may have been due to the use of unsuitably hard wood (as pigs interact more often with softer types of wood [18]). Some of these materials may have been recommended because they are known to be manipulated by pigs substantially. However, interaction with an enrichment does not necessarily mean that tail damage is reduced (as discussed further in Section 5). As such, there is a need for more detailed studies on these materials’ ability to reduce tail damage. In addition to suggesting specific types of enrichment, the EU recommends that (a combination of) enrichment should allow the pig to eat/smell, bite, and root it, and change its location, appearance, or structure [26]. In line with these recommendations, most of the enrichments that we identified as effective could satisfy at least some of these criteria. However, information on how pigs actually interact with enrichment is necessary to see if criteria are truly satisfied (see Section 6). Furthermore, some enrichments (mezzanines, hiding walls) satisfy none of the criteria, but were found to have a beneficial effect.

Most studies have applied enrichment preventively, but the efficacy of enrichment to curtail tail biting outbreaks has also been studied [3,45,46]. In commercial practice, adding enrichment once tail biting starts is relatively common [1]. An earlier study on the addition of enrichment did not include a non-intervention control group [42], but a more recent study showed that providing haylage (or straw) after the first wounded pig was observed significantly reduced the risk of a full tail biting outbreak. A rope with a sweet block tended to reduce the chance of a full outbreak [45]. In another study, intervening by adding rope did not lead to a significantly greater reduction of the risk of a full outbreak than adding a hanging toy with plastic sticks, although the percentage of pens with a full outbreak was numerically much lower after rope intervention than toy intervention (34% vs. 62%). No control treatment without intervention was included, so both interventions may have had some beneficial effects [46]. The results of the intervention studies are very promising, as they allow targeted application of (extra) enrichment to the groups of pigs that need it the most. This requires early identification of the start of a tail biting problem. Apart from monitoring the incidence of wounding, a change in tail posture (holding the tail tucked close to the body) has been shown to be a useful early warning sign of a tail biting outbreak [47,48,49,50,51]. In this way, tail biting can be detected even before the first pig is damaged, allowing even earlier intervention. However, baseline levels of tail posture vary markedly between pens in the absence of an outbreak [52]. Therefore, it is the change in pigs keeping their tails tucked down, rather than the absolute number of pigs doing so, that needs to be monitored. This needs to be done frequently (more than 3x per week [45]) to intervene timely, but can be automated [53]. The usefulness of monitoring other types of behaviour to predict tail biting outbreaks has been studied as well (body posture, enrichment use, ear biting, feeding, social networks, activity) but this has not yet provided a reliable indicator [47,48,52,54,55,56,57,58,59].

## 4. Effects of Non-Straw Enrichment on Behaviour Directed towards Conspecifics and the Environment

Tail damage constitutes a serious welfare issue. But chewing and biting on the tail does not always lead to tail damage [36]. Furthermore, tail manipulation can be higher in pens without tail biting than in pens that had actual outbreaks of tail biting [60]. Even in the absence of severe damage, tail biting behaviour can be seen as problematic, either because it leads to discomfort in victims or because it is a sign of unsatisfied behavioural needs in the biters [61]. Therefore, studies that observed tail biting directly, rather than relying on the damage it causes, are of value and are therefore discussed in this section. Different types of tail biting have been suggested to result from different unsatisfied needs [61], and some studies have distinguished between damaging tail biting and non-damaging “tail-in-mouth” behaviour [46,62]. However, few studies have made this distinction as doing so requires more time (both to distinguish different behaviours and because more data will be required for analysis when these behaviours are scored separately). In fact, most studies combine manipulation of several different body parts into one category. As only three studies were identified in which tail biting or tail-in-mouth behaviour were scored separately from all other pen mate manipulations [34,40,46], conspecific manipulation is discussed as one category in this review. More detailed observations of how pigs manipulate tails in specific situations (e.g., do they suck, chew, pull or root on tails) could contribute to designing optimal enrichments to which these types of behaviour could be directed. The development of automated video surveillance techniques may make this process more feasible in the future [63].

Table 2 shows that pigs provided with different kinds of non-straw enrichment devoted 2–11% of their time to pen mate directed behaviour. The greatest reduction in pen mate manipulation was achieved by providing fodder beets after weaning [43] and hessian sacks both before and after weaning [34]. Pigs in pens with such enrichment showed a 71% and 46% reduction of pen mate manipulation compared to their control pens, respectively. Nonetheless, some pen mate manipulation still occurred even when the most successful enrichments were used, although this is not necessarily problematic, as only excessive pen mate manipulation is regarded as harmful. Silage [64], compost [35], weekly changing objects [36], access to a mezzanine floor [37] and even an empty overhead rack with a wooden frame [35] also reduced pen mate manipulation, but to a lesser extent (13–38%). In the case of silage, the smaller reduction in pen mate manipulation may also be due to the highly valued enrichment in the control treatment (straw). Furthermore, since the reduction in pen mate directed behaviour was based on one study per type of enrichment, reductions may also have been influenced by the wider aspects of the study (i.e., the number of enrichments per pig and their location, and other housing, management and genetic factors) and should thus be interpreted cautiously. The type of enrichment also influences how pig-directed and pen-directed behaviour develop over time [19].

In most cases, the enrichments that were effective at reducing pen mate manipulation correspond with those effective at reducing tail damage. However, fresh wood, rope and the presence of a hiding wall reduced damage but not pen mate manipulation. Furthermore, the effect of beets and empty overhead racks on tail damage have not been quantified. Again, mostly in line with results on tail damage, a variety of other tested objects (metal, rubber, cloth, feed blocks, compressed shavings, and wood) had no significant effect on pen mate manipulation. Two studies that evaluated wood found that, contrary to intentions, it significantly increased pen mate manipulation [14,65], but not tail biting specifically [11]. This may be because enrichment can stimulate pigs to perform behaviours that they cannot direct to the enrichment in a satisfactory way, leading to frustration and increased re-direction of behaviour to pen mates [60]. 

A high level of manipulation of the pen’s components and its floor, rather than of the enrichment, is seen as a sign that the provided enrichment is not satisfactory [40]. Based on older literature, Beattie et al. [35] reported that the proportion of time that pigs in barren pens spent rooting on pen mates and pen fixtures was similar to the proportion of time that pigs in enriched environments spent rooting substrates that were considered more suitable. However, their own data suggest that access to compost led to much more interaction than the time spent on pen-directed and pig-directed behaviour in the absence of the enrichment [35]. Similarly, pigs were found to interact more with ‘substrates’ (defined to include not only enrichment but also pen components and other pigs) when enrichment was present [66]. This is in line with a recent meta-analysis showing that pigs with enrichment objects spent more time manipulating the floor than those without objects, especially at higher space allowances [67]. This may indicate that in some cases, interacting with objects increases the motivation to manipulate rather than diffusing it. 

Similarly to what was noted for pig-directed manipulation, several types of pen-directed manipulation are generally lumped into one category. As pigs interact with their pen in different ways (e.g., rooting the floor, biting pen components or scratching their body on these) further separation of different types of behaviour would facilitate the designing of novel types of enrichment. Pen-directed behaviour was significantly reduced by the provision of compost, silage, rope and the presence of a hiding wall (Table 2). The lower amount of enrichments which significantly reduced pen directed behaviour (compared to those reducing tail damage or pig-directed behaviour) is partly due to the lower number of studies including pen-directed behaviour. However, several studies on other types of enrichment included pen-directed behaviour but could not show a significant effect (metal, plastic and wooden objects, division of space, feed blocks/dispensers). 

## 5. Time Spent in Interaction with Non-Straw Enrichment

The percentage of time spent in interaction with an enrichment is commonly monitored when evaluating its suitability. Although it is often expected that more time spent in interaction with the enrichment will be associated with a decrease tail damage [16], this is not necessarily always the case. Co-occurrence of high enrichment use and tail biting has been reported in several studies. For instance, pigs that were provided with an overhead rack with which they interacted for 8% of their time had a higher incidence of tail damage than control pigs without enrichment (22 vs. 10%, [35]). In one study, pigs provided with more popular enrichment spent as much time manipulating their pen mates as pig provided with less popular enrichment [69]. In two further studies, pigs provided with more popular enrichment spent even more time manipulating their pen mates than pigs provided with less popular enrichment [36,43]. Although in some cases, this may have been caused by competition over popular enrichments, this cannot explain other cases where pigs spent an equal amount of time interacting with different enrichments but showed marked differences in tail damage [32]. Thus, it seems that interacting with certain types of non-straw enrichment may provide a more efficient outlet for stress or motivated behaviours that might otherwise be redirected towards conspecifics’ tails. 

As the proportion of the daily time budget that is spent on interaction with enrichments is generally relatively low (Table 3) and tail biting is not necessarily a highly time-consuming process, it seems unlikely that enrichment could prevent tail biting simply by keeping pigs occupied. Even when taking into account that pig spend a large proportion of their time inactive, apparently resting, this still leaves a considerable amount of time that could be used for tail biting (inactivity is usually estimated between 60–80% [64,65,66,68,70], although estimates as low as 24% are reported [71]). Furthermore, inactivity may itself be a response to lack of stimulation, as pigs spent more time inactive but alert in unenriched conditions than in enriched ones [72] and boredom may build up during such periods. In contrast to the low time spent in interaction with most enrichments, pigs interacted with scented ropes for a considerable amount of time (17%), and spent over 20% of their time interacting with roughage in some studies [64,73]. This comes closer to the proportion of time that pigs spend rooting under semi-natural conditions (1/3 of daylight hours, [35]) and is more likely to represent sufficient occupation. However, other studies on roughage manipulation reported rates of manipulation as low as 1% [43], in which case a reduction in tail biting by keeping the pigs otherwise occupied seems highly unlikely.

Table 3 shows how much time pigs spent interacting with different types of non-straw enrichment. Up to a 20-fold difference was observed between the most and least used enrichment within a study [66]. Not all studies presented their data in a way that can be recalculated in a percentage of time per pig. From those that did, it can be concluded that compost, peat, bark, silage and rope were able to occupy pigs more than 10% of their time in at least some of the studies [35,64,65,66,71,72,73], whereas plastic, metal or wooden objects only occupied pigs for 4% of their time at most [65] and generally less than 2% [40,68,74,75]. 

It is not just the type of non-straw enrichment that will predict its use, as shown by large differences in interaction with similar types of enrichment in different studies (Table 3). In addition to an influence of the wider contexts of the studies (i.e., differences in the quantity and location of enrichment and housing, management and genetic factors between studies), inconsistencies between studies in the timing of the observations and the definition of interaction likely contribute to such differences. For instance, objects that stimulate most interaction on the day of introduction do not necessarily receive sustained interest [28]. Nonetheless, meaningful influences on enrichment use can be discerned from the results summarized in Table 3 as well. When more enrichments are provided, this increases the total interaction [70,76], even if the enrichments are seemingly identical and are not used at such a high frequency that lack of access is likely to affect use. A meta-analysis [67] and recent study [32] also found that interaction with enrichment increased when different kinds of objects were provided. Furthermore, spreading enrichment over the pen allows pigs to choose their preferred location, which seems to boost use [76]. These results contrast with earlier findings that increasing the number of suspended crosses of flexible plastic did not significantly affect their use [75]. This suggests that adding more units may be more useful for certain types of enrichment than for others (e.g., those that are easily monopolized), or when additional units are placed in specific locations. Enrichment manipulation is also dependent on the situation in which it is applied. For instance, pigs manipulated maize silage more if stocked at a lower density [64] and manipulated a feeding tube more if fed a restricted diet [77]. Furthermore, the presentation of the enrichment contributes to the observed differences in usage. Although wooden objects in general did not occupy pigs for a high percentage of their time, a wood block elevated from the floor on one side by a plastic ring that could be moved through the pen was in use by at least one pig for 65% of the time (13.5 pigs/pen), indicating that this may be a more suitable way of providing wood than suspending it or mounting it to the wall [78]. In the same study, a set of spring-mounted plastic balls attached to the floor in a close triangular pattern were also occupied frequently (33% of the time by at least 1 pig). Both of these objects (wood block and spring mounted balls) were occupied more often than suspended objects intended for chewing. The key to this may be that these objects allow (partial) rooting patterns, as the pig uses the snout to move the block and balls. This potentially provides pigs with a possibility to ‘root’ when housed on fully slatted floors that hinder the provision of a sufficient amount of truly rootable substrate (e.g., compost, peat, bark, silage). However, the information on such rootable objects and the extent to which they truly satisfy the pig’s rooting motivation is extremely limited and further research is required. Furthermore, a reduction in the manipulation of floor-based enrichments due to soiling is a concern [67]. However, the aforementioned high use of the spring-mounted balls and the woodblock with ring, and an absence of an effect of regular cleaning of such devices [78] indicates that soiling is not necessarily a problem for all floor-based devices. Lastly, any enrichment that is inaccessible to the pig, e.g., items suspended too high or biting substrates that are too large for the pig to fit its mouth around, will obviously not be effective [5].

It is well known that pigs’ interest in many types of non-straw enrichment decreases rapidly in the first few days after its introduction [12,46,65,78]. It has been recommended to restrict exposure to each object to less than 2 days to preserve its exploratory value [79]. However, this would require more than 80 different objects to sustain interest throughout the pig’s life, and re-using objects several times in a rotation scheme seems inevitable. When enrichment devices were changed weekly, pigs interacted less with an enrichment they encountered for the second time 7 weeks later than when they were exposed to it for the first time [36]. Nonetheless, one study found a tendency for a reduction in tail damage when enrichment was rotated every two weeks, even if they did not observe a difference in interaction with the enrichment [80].

In addition to evaluation of enrichment use in the home pen, pigs’ motivation to interact with different types of enrichment has been evaluated in preference tests. Pigs showed a preference for short-term (3 min) interaction with peat or compost over wood shavings in a three-choice maze test [78]. Within the other tested non-straw categories, no clear preferences could be determined (fir chips vs. willow chips vs. spruce chips, sugar beets vs. maize silage vs. grass silage, alfalfa hay vs. seed grass vs. barley grass, and a suspended bite-rite toy vs. sisal rope vs. wood). In the last category, pigs chose to remain in the centre of the maze more often than to enter any of the rooms where they could interact with the enrichment, suggesting that all these materials are of minimal interest, at least when provided in a relatively unknown arena that may be interesting to the pigs itself. Another study showed that pigs preferred sand with carrots or just carrots over just sand, whilst valuing just carrots and sand with carrots equally [83].

## 6. Type of Interaction with the Enrichment

Pigs can interact with environmental enrichment in a variety of ways and different types of enrichment are likely to stimulate different activities. Consultation amongst welfare scientists and previous literature analysis have indicated that not only the amount, but also the method of interaction is important when assessing the suitability of enrichment [29]. Apart from the degree to which the enrichment allows exploration and learning, its suitability for rooting was regarded as the most important characteristic. Rooting is a high priority behaviour in pigs [21,83]. Tail biting is suggested to be a redirection of rooting behaviour to pen mates in the absence of suitable rooting substrates [35], although the multifactorial nature of tail biting has also been emphasized and a redirected rooting motivation may be only one of many underlying causes of tail biting [61]. Although pigs prefer rooting materials containing feed, rooting is considered an intrinsically motivated behaviour that is performed irrespective of its original function in feed seeking behaviour [35,83]. Ringed pigs, which are unable to root, will explore more by chewing, sniffing and manipulating rooting materials whilst unringed pigs choose to root these. The prevention of rooting by ringing did not lead to abnormal behaviour unless these other ways of exploring were also prevented [21]. This suggests that although rooting can be partially replaced by other behaviours, this is not what pigs prefer. Allowing the pig to perform the preferred type of exploratory behaviour or to use its preferred rooting substrate may satisfy the pig’s specific motivations to a greater extent [83]. This, in turn, would be expected to have a greater impact on the redirection of rooting behaviour towards pen mates. In addition to rooting, the suitability for nosing and biting were also considered important by the welfare expert panel, but pushing, pulling, shaking, carrying and chewing less so [29]. As such, it seems highly relevant not only to evaluate how much pigs interact with different types of enrichment, but also how they interact with it.

Rather than basing a judgement of an enrichment’s suitability for rooting on observation of this behaviour, enrichments are usually labelled as rootable based on pre-assumptions (e.g., [28]). Information on the time actually spent rooting enrichments is scarce because many studies have lumped several different behaviours including use of the snout under one term (either rooting or nosing). However, a clear separation was made by Day et al. [68], who defined rooting as substrate displacement by circular snout movements. They report that pigs never rooted, but exclusively chewed, on a chain. Interactions with a flavoured bar included some rooting (less than a fifth of the interaction time), but (near) nasal contact and chewing were more common. Haskell et al. [66] used a slightly different definition of rooting (nudging or lifting substrate with the top of the rooting disc, with substrate defined as straw, bark, branches, faeces, pigs, or pen components). They found that only 1% of the interaction time was spent rooting, whereas chewing and touching the substrate with the nose were far more common. In another study, hessian sacks hanging from the pen wall were bitten 8 times as often as manipulated with the nose [34]. Although some of these types of enrichment can be said to be little suited to rooting, rooting has also been reported to take up only a minimal part of the interaction time with straw (less than 1/13) [68]. These studies show that rooting (defined as moving or lifting substrate with the nose) is only minor component of interaction with enrichment. This could mean that the pig’s motivation to root is overestimated, that the types of enrichment tested were not sufficiently suited to rooting behaviour, or that although rooting is highly motivated, this motivation is rapidly satisfied when given the right substrate. Crucially, it also means that measuring the time of interaction only is unlikely to be a good estimate of the extent to which an enrichment actually satisfies a pig’s rooting motivation. 

Chewing may reflect the motivation to feed, to explore, or both [77]. This makes the interpretation of interaction with chewable enrichments complex. Chewing is a main way of deforming objects, and deformable objects are manipulated more than non-deformable ones [12,67]. As deforming an object introduces novelty, this supports the involvement of exploratory motivation in enrichment chewing. On the other hand, pigs interacted more with enrichment when fed restrictively [69,77] or fed a diet with inadequate crude protein [84], suggesting that a motivation to feed can affect pigs’ interaction with enrichment. However, in contrast to restrictively fed pigs, ad libitum fed pigs showed no preference for an enrichment providing a sucrose solution (sweet tasting solution containing energy) over one providing a saccharine solution (sweet tasting solution without energy) [77]. This suggests that the advice to use chewable enrichments with a nutritional benefit [26] is mainly relevant for pigs that are not fed ad libitum (which in commercial practice is limited to gestating sows, the end stages of pigs reared to heavy slaughter weights, and to fast growing breeds to avoid excessive fat deposition). Moreover, if pigs interact more with an enrichment device with a nutritional benefit because it satisfies a feeding motivation, this would not necessarily be expected to affect behaviour redirected to pen mates. Whilst it is commonly assumed that exploratory behaviour can be redirected at pen mates [21,77], it seems unlikely that the same is true for feeding motivation because recent studies [25,85,86,87] show minimal or no effects of restricted feeding or fibre inclusion on tail biting. Marked exceptions to this are the delayed arrival of feed [25] and insufficient feeder space [1]. However, these are more likely to represent a frustration of expected patterns and competition over access than an actual feeding motivation. In addition to the effect of feed availability, there might be a role of dietary crude protein and amino acid profiles (tryptophan, methionine, threonine) on tail directed behaviour. However, a connection between such factors and tail damage was suggested rather than proven in such studies [84,88,89].

How a pig can use an enrichment object does not only depend on its material, size and shape, but also on its presentation. A meta-analysis suggested that objects attached to the floor are less suitable than suspended ones [67], but this may be due to confounding between the type of enrichment and where it is supplied. Others have argued that floor-mounted objects can be manipulated more vigorously than suspended ones, allowing a more satisfactory performance of rooting and chewing behaviours [78]. It has also been argued that suspended devices may be difficult for the pig to grip and can therefore cause frustration [30,90]. In line with this, items mounted or placed onto the floor were manipulated more by pigs than suspended chewing devices of plastic and metal [78]. However, objects that are provided loose on the floor may be unsuitable for vigorous manipulation unless they are heavy, thus requiring effort to push or pull across the floor. In this review, we identified both suspended and floor-based enrichment that effectively reduced tail damage or pen-mate directed behaviour (Table 1 and Table 2). Thus, although the presentation methods may affect the way the pig can use the enrichment, both presentations can contribute to the reduction of unwanted behaviour. Colour [91] and orientation of suspension (horizontal or vertical) [81] do not seem to affect enrichment use, although data on this is very limited.

EU recommendations specify that pigs should be provided with enrichment that they can eat/smell, bite, root. Furthermore, the pig should be able to change the location, appearance, or structure of this enrichment [26]. With the exception of a limited number of bedding materials which are expected to satisfy these criteria on their own, the use of a combination of different enrichments is advised to meet all criteria. However, the number of experiments in which these different forms of interaction were actually studied separately is extremely limited. Thus, there is a need for more detailed information on how pigs interact with different types of enrichment, rather than whether they interact with them. Ideally, such studies would also draw conclusions on how the different types of interaction influence tail biting behaviour, as interacting with the enrichment in a specific way does not necessarily reduce the pig’s motivation to direct this behaviour to other pigs [60].

## 7. Conclusions 

There are several kinds of non-straw enrichment that effectively contribute to the reduction of tail damage, either when applied preventively or when introduced at the first signs of tail biting. Effective materials include roughage, hessian sacks, compost, freshly cut wood, horizontal and vertical space dividers, rope, and weekly exchanged objects. No evidence was found that processed wooden, plastic, or metal objects that are not exchanged regularly reduce tail biting. This is worrying as these are commonly applied to this end in practice. Newer, potentially more effective objects are being introduced, but their effectiveness remains to be shown. Although recommendations on enrichment emphasize the need for several modes of interaction with the enrichment, only a limited number of studies has actually observed in detail how pigs interact with enrichment. This not only makes it difficult to comply with such recommendations, but also raises doubts about their scientific basis. Similarly, studies on enrichment have often pooled different manipulations of other pigs, and of the environment, into broad categories. More detailed observation of how pigs interact with different kinds of enrichment and which behaviours are directed at pen mates and the environment in their absence, would improve our understanding of how to combine enrichments for a maximum effect on tail biting. This is essential because although single enrichments can reduce tail biting significantly, the remaining levels of damage can still be high in undocked pigs. Thus, moving away from tail docking in a way that improves pig welfare requires more effective enrichments or better control over the other factors that influence tail biting.

## Figures and Tables

**Table 1:** Effects of environmental enrichment on tail damage ordered by type of enrichment (most successful types at the top of the table).

Enrichment	Other Enrichments Present	Fold Change						Description If ‘Other’	Means (Enr. vs. Ctrl)		Doc-Ked?	Stage	Ref
		Severe Dam.		Mild Dam.		Other			Sev. Dam.	Mild Dam.			
**Roughage**													
Suspended haylage ball (650 g/pen/day) at 1st wound	Suspended wooden sticks, straw	-		-		**0.12**	*	% pens >3 wounded pigs	-	-	No	W	[45]
Hay (60 g/pen twice daily)	Plastic & wooden objects (+ extras if damage occurred)	-		-		**0.92**	*	% pigs with partial tail loss	-	-	No	All	[33]
Corn silage (50 g/pen twice daily)	Plastic & wooden objects (+ extras if damage occurred)	-		-		**0.84**	*	% pigs with partial tail loss	-	-	No	All	[33]
**Fabric**													
Wall-mounted hessian sacks pre- & post-weaning	Chain, plastic toy	**0.19**	*	-		-		-	3 vs. 16%	-	Yes	≤13 weeks	[34]
**Separation of space**													
Mezzanine (adding 0.25 m^2^ extra space)	Chain	∞	*	**0.67**	*	-		-	0 vs. 2%	2 vs. 3%	Yes	G-F	[37]
Hiding wall	Chain	∞	*	∞	*	-		-	0 vs. 2%	0 vs. 4%	No	G-F	[39]
**Earth-like**													
Compost in overhead rack	No	**0.09**	*	-		-		-	1 vs. 10%		^?^	G-F	[35]
**Multiple**													
Suspended rope + paper prior to weaning only	Pre-wean: shavings + ball Post-wean: rope + plastic toy	**0.31**	*	**1.31**	*	-		-	10 vs. 32%	59 vs. 45%	No	W	[38]
Weekly changing objects ^2^	Wall-mounted chain	**0.30**	*	**0.58**	*	-		-	1 vs. 2%	2 vs. 4%	Yes	W +G-F	[36]
Plastic pipe + branched chain + fresh wood	Straw, wood shavings, chain	0.79	^NS^	**0.58**	*	-		-	22 vs. 28%	22 vs. 38%	No	G-F	[32]
Suspended rope with sweet block, end on floor, at 1st wound	Suspended wooden sticks, straw	-		-		**0.38**	^#^	% pens >3 wounded pigs	-	-	No	W	[45]
**Wood**													
Horizontally suspended fresh wood	Straw, wood shavings, chain	1.07	^NS^	**0.39**	*	-		-	30 vs. 28%	15 vs. 38%	No	G-F	[32]
Horizontal, frame mounted post	Chain (control group only)	1.31	^NS^	0.47	^NS^	-		-	17 vs. 13%	14 vs. 30%	No	W	[40]
Horizontal cylinder of compressed wood shavings	Chain (control group only)	∞	^NS^	1.00	^NS^	-		-	0 vs. 5%	18 vs. 18%	No	W	[40]
**Alternative feeding**													
Pressed feed block, fixed to frame but rotatable	Chain (control group only)	∞	^NS^	0.72	^NS^	-		-	0 vs. 5%	13 vs. 18%	No	W	[40]
Pellets scattered on the floor	Chain, plastic ball	-		-		^?^	^NS^	Tail damage score	-	-	Yes	G-F	[41]
**Suspended metal**													
Cross of chains	Straw, wood shavings, chain	0.57	^NS^	1.05	^NS^	-		-	16 vs. 28%	40 vs. 38%	No	G-F	[40]
Overhead rack		2.18	^1^	-		-		-	22 vs. 10%		^?^		[35]
**Suspended plastic/rubber**													
Cross of polythene pipes	Straw, wood shavings, chain	0.61	^NS^	1.05	^NS^	-		-	17 vs. 28%	40 vs. 38%	No	G-F	[32]
Cross of rubber hose pipes	Chain (control group only)	-		-		0.93	^NS^	% pens ≥1 wounded pig	-	-			[42]
**Variable** ^3^													
Chains or objects	Variable	0.88	^NS^	-		-		-	-	-	Both	G-F	[23]
Chains, plastic objects, wood, or rooting material	No	-		-		1.61	^NS^	% farms ≥1 wounded pig	-	-	Yes	W	[25]
Chains, plastic objects, wood, or rooting material	No	-		-		0.86	^NS^	% farms ≥1 wounded pig	-	-	Yes	G-F	[25]

Severe dam.: severe tail damage (partial or complete tail loss, crust formation, infection, fresh blood, or clearly visible wounds); Mild dam.: mild tail damage (small bite marks, superficial bites or scratches, tail-end hair missing, or blood on the tail); ∞: Infinite (i.e., damage reduced to 0); * Significant difference between enriched and control group within a study (*p* < 0.05); ^#^ Tendency towards a significant difference between enriched and control group within a study (*p* < 0.10); ^NS^ No significant difference between enriched and control group within a study (*p* > 0.05); W: Weaners, G-F: Growers/fatteners; ^1^ Pairwise significance not clearly identified; ^2^ Rubber bar, rubber ball, yellow ribbon, rope, yellow rubber hose, purple ribbon, grey garden hose, attached to the wall mounted chain; ^3^ Indicates that a different material was applied in different groups, but these were analysed as one category; ^?^ Not reported.

**Table 2:** Effects of enrichment on manipulation of pen mates and pen components.

Enrichment	Manipulating Pigs			Manipulating Pen/Floor			Other Enrichments Present	Doc- ked?	Stage	Ref
	% Time ^1^	Fold Change ^2^		% Time ^1^	Fold Change ^2^					
Chopped fodder beet	-	0.29	*	-	-		Straw bedding + box	^?^	G-F	[43]
Wall-mounted hessian sacks pre- & post-weaning		0.54	*	-	-		Chain, plastic toy	Yes	W + G-F	[35]
Hay of grass and clover	-	0.55	^NS^	-	-		Straw bedding + box	^?^	G-F	[43]
Weekly changing objects ^3^	-	0.62	*	-	-		Wall-mounted chains	Yes	W + G-F	[36]
Compost in overhead metal rack with wooden frame	4.0	0.66	*	6.0	0.86	*	No	^?^	G-F	[35]
Whole-crop silage of barley and peas	-	0.68	^NS^	-	-		Straw bedding + box	^?^	G-F	[43]
Green grass meal	-	0.71	^NS^	-	-		Straw bedding + box	^?^	G-F	[43]
Suspended chains, wall-mounted bar, loose tyres	3.0	0.75	^NS^	-	0.95	^NS^	No	^?^	G-F	[11]
Mezzanine floor adding 0.25 m^2^ extra space	2.6	0.75	*	12.2	0.95	^NS^	Chain	Yes	G-F	[37]
Maize silage	2.2	0.77	*	3.3	0.73	*	Chopped straw (control only)	Yes	G-F	[64]
Whole-crop silage of clover and grass	-	0.79	^NS^	-	-		Straw bedding + box	^?^	G-F	[43]
Two sets of objects swapped each week	3.0	0.85	^NS^	-	1.32	^?^	No	^?^	W + G-F	[10]
Suspended plastic sticks fixed to a central plastic cone	-	0.86	^NS^	-	1.04	^NS^	No ^1^	No	G-F	[60]
Flavoured bar in a dispenser with drinker and trough	2.0	0.87	^NS^	5.0	0.86	^NS^	No	Yes	G-F	[68]
Overhead metal rack with wooden frame	5.0	0.87	*	7.0	1.00	^NS^	No	^?^	G-F	[35]
Horizontal cylinder of compressed wood shavings	4.1	0.87	^NS^	1.4	1.17	^NS^	Suspended chain (control only)	No	W	[40]
Pressed feed block, fixed to frame but rotatable	4.2	0.89	^NS^	1.6	1.33	^NS^	Suspended chain (control only)	No	W	[40]
Suspended rope	8.1	0.95	^NS^	7.1	0.79	*	Wood block on floor, washed daily	Yes	W	[65]
Horizontal frame mounted wooden post	5.1	1.00	^NS^	3.0	0.86	*^6^	Suspended chain (control only)	No	W	[40]
Suspended looped chain	2.0	1.05	^NS^	6.0	1.13	^NS^	No	Yes	G-F	[68]
Suspended hard wood (in larger high density groups)	10.3	1.11	*^5^	9.3	1.18	^NS^	No	Yes	G-F	[14]
Hiding wall	2.8	1.12	^NS^	8.7	0.79	*	Suspended chain	No	G-F	[39]
Dispenser that released feed upon manipulation	-	1.14	^NS^	-	1.00	^NS^	No ^7^	No	G-F	[60]
Wood block placed on floor (washed daily)	8.1	1.17	*	7.1	1.15	^NS^	Suspended rope	Yes	W	[65]
Whole-crop silage of oats, vetch and lupine	-	1.26	^NS^	-	-		Straw bedding + box	^?^	G-F	[43]
Suspended hard wood (in smaller low density groups)	11.1	1.52	*^5^	11.0	1.20	^NS^	No	Yes	G-F	[14]
Flavoured water dispenser (prior to experiment)	-	2.00	*	-	-		Plastic sticks fixed to a central cone	No	G-F	[60]
Fresh wood, chains, plastic pipes separately or together	-	-	^NS^	-	-		Straw, wood shavings, chain	No	G-F	[32]

* Significant difference between enriched and control groups within the study (*p* < 0.05); ^NS^ No significant difference between enriched and control groups within the study (*p* > 0.05); W: Weaners, G-F: Growers/fatteners; ^1^ % of time per pig in groups with access to the enrichment. Depending on the original data this percentage could be based on continuous observation of focal animals or scan sampling of a known number of individuals (but not on frequencies of behaviour obtained from continuous observations, or the percentage of scans in which the behaviour was performed by an unspecified number of individuals). Sometimes it was not possible to recalculate data into the percentage of time due to the sampling protocol used or insufficient information about this protocol; ^2^ Level in enriched groups/level in control groups; ^3^ Rubber bar, rubber ball, yellow ribbon, rope, yellow rubber hose, purple ribbon, grey garden hose, attached to the wall mounted chain; ^5^ Although enrichment increased pen mate manipulation in total, tail biting was decreased by enrichment; ^6^ Both floor and pen manipulation affected, but in different directions; ^7^ A non-functional fluid dispenser was present in control pens only; ^?^ Not reported.

**Table 3:** Interaction with different types of enrichment compared within one study.

Enrichment	Fold Change from Least Used Enrichment ^1^	% of Pig’s Time Spent in Interaction ^2^	% Time Enrichment in Use	% of Minutes Including Interaction	Stage	Ref.
Maize silage	1.23	23	-	-	G-F	[64]
Suspended garlic scented rope Suspended rope	4.00	17 4	-	-	W	[71]
Peat (6 cm deep layer, 2.8 m^2^)	-	15	-	-	W + G-F	[72]
Bark	20.00	14	-	-	G-F	[66]
Branches	1.00	0.7	-	-		
Rope and wood provided continuously	3.62	13	-	-	W	[65]
Weekly alternation between 1: wood and 2: rope	2.97	11	-	-		
Rope suspended from a wall bracket	2.76	10	-	-		
Weekly alternation between 1: rope and 2: wood	2.51	9	-	-		
Wood block placed on floor (washed daily)	1.00	4	-	-		
Compost in overhead rack	1.63	13	-	-	G-F	[35]
Empty overhead rack	1.00	8	-	-		
Sucrose solution tube (energy restricted pigs)	3.85	7	-	-	G-F	[77]
Saccharin solution tube (energy restricted pigs)	1.85	4	-	-		
Water tube (energy restricted pigs)	1.00	2	-	-		
Turf	2.33	4	-	-	W	[74]
Peat filled tray	1.00	2	-	-		
Suspended rope	11.57	4	-	-	W	[74]
Artificial dog bones	4.73	1	-	-		
Suspended chains	1.00	0.3	-	-		
Suspended hard wood (in larger high density groups)	-	4	-	-	G-F	[14]
Suspended hard wood (in smaller low density groups)	-	4	-	-	G-F	
Sucrose solution tube (non-energy restricted pigs)	1.38	3	-	-	G-F	[77]
Saccharin solution tube (non-energy restricted pigs)	1.08	2	-	-		
Water tube (non-energy restricted pigs)	1.00	2	-	-		
4× cross of flexible plastic piping suspended on chain	1.56	1.4	-	-		[75]
1× cross of flexible plastic piping suspended on chain	1.00	0.9	-	-		
Silage of oats, vetch and lupine roughage box	6.37	1.2	-	-	G-F	[43]
Chopped fodder beet in roughage box	2.17	0.4	-	-		
Whole-crop silage of clover and grass in roughage box	1.97	0.4	-	-		
Whole-crop silage of barley and peas in roughage box	1.75	0.3	-	-		
Green grass meal in roughage box	1.33	0.3	-	-		
Hay of grass and clover in roughage box	1.06	0.2	-	-		
Empty roughage box	1.00	0.2	-	-		
Horizontal cylinder of compressed wood shavings	1.58	0.8	-	-	W	[40]
Suspended metal chain	1.08	0.5	-	-		
Pressed feed block, fixed to frame but rotatable	1.00	0.5	-	-		
Suspended metal chain	1.16	0.7	-	-	W	[40]
Horizontal frame-mounted wooden post	1.00	0.6	-	-		
4× wooden beams in racks, could be moved vertically	3.14	0.6	-	-	G-F	[57]
2× wooden beams in racks, could be moved vertically	1.00	0.2	-	-		
Flavoured bar in a dispenser with drinker and trough	8.20	0.4	-	-	G-F	[68]
Suspended looped chain	1.00	0.1	-	-		
Multiple suspended wooden sticks	2.57	0.2	-	-	G-F	[70]
Wooden stick fixed to wall, could be moved vertically	1.43	0.1	-	-		
Suspended wooden stick	1.00	0.1	-	-		
Wooden block, elevated on one side by a plastic ring	9.29	-	65	-	G-F	[78]
Ropes fixed to chains movable within an elevated pipe	5.71	-	40	-		
Triangular assembly of spring-mounted plastic balls	4.71	-	33	-		
Suspended scented plastic ring	2.86	-	20	-		
Plastic sticks fixed to a suspended central cone	2.29	-	16	-		
Suspended disc with plastic strips and chains	1.57	-	11	-		
Rigid plastic ball, loose on floor	1.00	-	7	-		
Suspended knotted rope	2.29	-	32	-	G-F	[78]
Triangular assembly of spring-mounted plastic balls	1.79	-	25	-		
Rigid plastic balls, loose on floor	1.21	-	17	-		
Plastic sticks fixed to suspended central cones	1.00	-	14	-		
Rubber dog toy suspended on an elastic cord	4.46	-	31	-	W	[12]
Knotted rope suspended on an elastic cord	1.71	-	12	-		
Rubber hose suspended on an elastic cord	1.29	-	9	-		
Metal chain suspended on an elastic cord	1.00	-	7	-		
Rope (suspended horizontally or vertically)	3.59	-	8	-	G-F	[81]
Metal pipe (suspended horizontally or vertically)	1.41	-	3	-		
Wood (suspended horizontally or vertically)	1.32	-	3	-		
Chain (suspended horizontally or vertically)	1.00	-	2	-		
Wall mounted metal chain with rubber hose around it	2.00	-	-	8	W +	[82]
Rubber hose, placed on floor	1.75	-	-	7	G-F	
Rope, suspended from spring	1.75	-	-	7		
Hard plastic ball with stones inside, loose on the floor	1.00	-	-	4		
Chopped myscanthus, provided on floor	1.20	-	-	6	G-F	[69]
Bark compost in a floor level trough	1.00	-	-	5		
Feed dispenser	3.00	-	-	-	G-F	[60]
Plastic sticks fixed to a suspended central plastic cone	2.40	-	-	-		
Non-functioning liquid dispenser	1.00	-	-	-		
Rope suspended from wall-mounted chain	1.70	-	-	-	W +	[36]
Purple ribbon suspended from wall-mounted chain	1.50	-	-	-	G-F	
Yellow ribbon suspended from wall-mounted chain	1.40	-	-	-		
Grey hosepipe suspended from wall-mounted chain	1.25	-	-	-		
Rubber bar suspended from wall-mounted chain	1.20	-	-	-		
Rubber ball suspended from wall-mounted chain	1.10	-	-	-		
Yellow hosepipe suspended from wall-mounted chain	1.00	-	-	-		
Combination of all materials below	6.20	-	-	-	G-F	[32]
Suspended plastic pipes + single chain	5.80	-	-	-		
Suspended freshly cut wood + single chain	5.40	-	-	-		
Suspended branched chains end on floor + single chain	1.20	-	-	-		
Suspended single chain	1.00	-	-	-		

Within the category of output variable, studies have been ordered according to the level of interaction with the most used enrichment; ^1^ Fold change: level of interaction with an enrichment / level of interaction with the least used enrichment in that study. Note that the type of least used enrichment varies between studies, limiting the validity of comparing fold changes across studies; ^2^ % of time per pig. Depending on the original data this percentage could be based on continuous observation of focal animals or scan sampling of a known number of individuals (but not on frequencies of behaviour obtained from continuous observations, or the percentage of scans in which the behaviour was performed by an unspecified number of individuals); W: Weaners, G-F: Growers/fatteners.
